# Two Cases of Puerperal Peritonitis

**Published:** 1854-01

**Authors:** James P. White

**Affiliations:** Professor of Obstetrics in Buffalo Medical College; Buffalo


ART. III.—*Two Cases of Puerperal Peritonitis.* Treated by Opium at the Lying-in-Ward of the Buffalo Hospital of the Sisters of Charity. By JAMES P. WHITE, M. D., Professor of Obstetrics in Buffalo Medical College.

CASE I. Elizabeth Wilson, aged 22 years, entered the hospital in July, expecting to be confined in September. Is unmarried—has been seduced by promise of marriage—is very desponding. Quickened May 1st. Fœtal heart in left iliac region—a left occipito-anterior presentation is predicted.

*Sept.* 12, 1853. Membranes ruptured at M. At 5, P. M., pains were sharp; occiput presenting in the left anterior position. At 7, P. M., the pains were so severe that chloroform was administered, and its influence kept up during each pain with much relief. At 8, 30, P. M., was delivered of a large boy, weighing 10¾ lbs. At 10, P. M., took 8 grs. Dover's powder; the pulse was at that time 108.

*Sept.* 13. Slept none from pain, which is very severe, in the abdomen. Countenance very much flushed; skin hot; some distention of abdomen from flatus; pulse 108.

℞ Pulv. Doveri, grs. viij.
Morph. sulph., grs. ¼, every four hours, and spts. nitre dulc. ℥j. every four hours, given two hours after the powder.

9, P. M. Pulse now 112. A fomentation of brandy, laudanum, and water, has been used during the day.

14*th,* 11, A. M. Pulse ranges from 118 to 130. There is great tympanitis. The pain is very severe when the uterus is pressed, but slight pressure does not cause pain; thus showing that the inflammation has not extended to the peritoneum lining the abdominal parietes.

℞ Morph. sulph. gr. ¼, every hour till on the verge of narcotism; also
Quiniæ sulph. gr. j, every two hours.
Continue the fomentations.

15*th.* Drs. Flint and White in consultation at 10, A. M. Slept well; is comparatively easy this morning. Face is pale and anxious; pulse 118 to 130.

℞ Increase the morphine to gr. $\frac{1}{3}$, every hour. Give also quinine, gr. j, every two hours; each to be taken in spts. nitre, dulc. ʒj.

16*th.* Drs. Flint and White in consultation. Pulse 118 to 124; abdomen not so much distended as on the evening previous.

℞ Morph. sulph. gr. $\frac{1}{2}$.
Quin. sulph. gr. jss. every two hours, in spts. nitre dulc. ʒj. Use the hop fomentation constantly; change it hourly, covering it with oiled silk.

18*th.* Pulse is now 100. She is quite easy.

℞ Quin. sulph. gr. xij.
Morph. sulph. gr. iv.
Spts. nitre dulc. ʒxij.

Give a teaspoonful every two hours.

At evening her pulse was 90.

19*th.* Pulse the same, feels much better.

21*st.* Pulse 86.

℞ Morph. sulph. gr. $\frac{1}{3}$, every four or five hours.

22*d.* Pulse 88. Some pain in rectum, but none elsewhere.

℞ Enema of water, Oj.

This brought away a very little fœces.

23*d.* Pulse is now 100, which is attributed to irritation in the rectum. There is no pain in the abdomen. The enema was repeated at evening, procuring two copious evacuations, which afforded much relief.

24*th*, A. M. Pulse is still 100, but patient is comfortable. At 6, P. M., the pulse had receded to 86.

25*th.* Pulse 84.

26*th.* Pulse 72. The patient is now sitting up some of the time, and convalescence is established.

Case II. Catharine Close, a German, aged 22 years, lost her husband on shipboard four months since.

*Sept.* 16. Was delivered by forceps, at 10, A. M., after seventeen hours labor. Dover's powder was given every four hours during the remainder of the day.

17*th*. Says she feels pretty well. Pulse 100, abdomen somewhat tender. Continue the Dover's powder and apply warm fomentations.

18*th*. Has had chills this morning. Pulse 110; tenderness increased.

℞ Morph. sulph. gr. $\frac{1}{8}$ in spts. nitre dulc. ʒj, every second hour, unless relieved.

19*th*, 9, A. M. Pulse 120 and soft; abdomen tympanitic and tender; respiration hurried; thirst urgent; lochia entirely suppressed, and breasts flaccid.

℞ Morph. grs. iv.
Spts. nitre dulc. ℥j. M.

Give a teaspoonful every two hours unless she is narcotized. Apply warm fomentations under oiled silk.

8, P. M. Pulse 130, soft and compressible; countenance anxious; perspiration copious. Give a teaspoonful of the following mixture every second hour:

℞ Quin. sulph. grs. xviij.
Morph. sulph. grs. vi.
Spts. nitre dulc. ℥jss. M.

Continue fomentations, with mustard to the epigastrium to allay retching, which now annoys her.

20*th*. Pulse 114 and feeble; abdomen greatly distended.

Give the morphia and quinine every hour unless narcotism is induced. Continue fomentations, and give her brandy and beef essence as she can bear it. Evening—Patient is a little heavy, though easily aroused. Suffers less; pulse the same.

21*st*. Has slept considerably; has taken the medicine and nutriment as directed. Pulse 140. Continue treatment.

22*d*. Has taken the morphine every hour; has slept nearly all the time, only as aroused to take her medicine and drinks. Pulse 130, with more firmness. Continue treatment.

23*d*. Pulse 120. Seems better; abdomen less distended, and tenderness diminished. Continue the anodyne and tonic every second hour, with nutriment as she can take it.

24*th*. Pulse 108, and general appearance greatly improved. Decrease the anodyne to $\frac{1}{2}$ gr. every four hours.

The patient now went on to recovery, and on the 30th the lacteal secretion was fully established, and she free from danger. It is worthy of remark that in neither of the foregoing cases were the bowels moved during the first week. One had a spontaneous movement on the eighth, and the other on the tenth day after the attack; but not until the tympany was greatly diminished, aud the tenderness relieved.

Two cases treated before the adoption of the anodyne plan, proved fatal.

BUFFALO, 6th December, 1853.

